# Enhancing the Thermal Conductivity of Epoxy Composites via Constructing Oriented ZnO Nanowire-Decorated Carbon Fibers Networks

**DOI:** 10.3390/ma17030649

**Published:** 2024-01-29

**Authors:** Wei Lin, Chang Yu, Chang Sun, Baokai Wang, Mengyang Niu, Mengyi Li, Weiwei Xuan, Qi Wang

**Affiliations:** 1 Institute for Advanced Materials and Technology, University of Science and Technology Beijing, Beijing 100083, China; linwei@ustb.edu.cn; 2 School of Materials Science and Engineering, University of Science and Technology Beijing, Beijing 100083, China; 3 School of Energy and Environmental Engineering, University of Science and Technology Beijing, Beijing 100083, China

**Keywords:** carbon fiber, thermal conductivity, freeze-casting, epoxy composites

## Abstract

With the miniaturization and high integration of electronic devices, high-performance thermally conductive composites have received increasing attention. The construction of hierarchical structures is an effective strategy to reduce interfacial thermal resistance and enhance composite thermal conductivity. In this study, by decorating carbon fibers (CF) with needle-like ZnO nanowires, hierarchical hybrid fillers (CF@ZnO) were rationally designed and synthesized using the hydrothermal method, which was further used to construct oriented aligned filler networks via the simple freeze-casting process. Subsequently, epoxy (EP)-based composites were prepared using the vacuum impregnation method. Compared with the pure CF, the CF@ZnO hybrid fillers led to a significant increase in thermal conductivity, which was mainly due to the fact that the ZnO nanowires could act as bridging links between CF to increase more thermally conductive pathways, which in turn reduced interfacial thermal resistance. In addition, the introduction of CF@ZnO fillers was also beneficial in improving the thermal stability of the EP-based composites, which was favorable for practical thermal management applications.

## 1. Introduction

With the continuous progress of electronic technology, chips and electronic devices are moving toward the direction of miniaturization and high power density, which brings a powerful use of functions, but also leads to serious heat dissipation problems [1,2,3]. If the heat generated by the chip cannot be eliminated in time, it will seriously affect the performance, reliability, and service life of electronic devices. Therefore, carrying out efficient thermal management of electronic devices has become one of the biggest challenges in the electronics industry. In the field of heat dispassion, thermal interface materials (TIMs) play an important role, generally used between chips and heat sinks to fill with the microscopic voids and holes, thus reducing the contact thermal resistance, establishing effective pathways for heat transfer, and improving the heat dissipation ability of electronic devices. Therefore, the development of high-performance TIMs is of great scientific significance and commercial value in response to the rapidly growing demand for heat dissipation [4,5,6,7].

Polymers have the advantages of good flexibility, high resistivity, and good chemical stability, which are widely used as the matrix for TIMs [8,9,10]. However, the low intrinsic thermal conductivity (<0.5 W·m^−1^·K^−1^) makes it difficult to meet the increasing heat dissipation requirements. The current common method of preparing TIMs is to add abundant fillers with high thermal conductivity to the polymer matrix. So far, a variety of thermally conductive fillers have been reported, such as metals (Al [11], Cu [12], Ag [13]), ceramics (AlN [14,15], Al_2_O_3_ [16], and BN [17,18]), and carbon materials (CF [19], carbon nanotubes [20], diamond [21], and graphene [22]). Carbon materials are widely used as fillers for TIMs due to their high thermal conductivity. Among all carbon materials, diamonds have excellent thermal conductivity and good dispersibility, but their high price strongly limits their commercial application. Graphene has an extremely high theoretical thermal conductivity and is one of the most widely reported fillers in recent years. However, the high anisotropy of graphene results in ultra-high in-plane thermal conductivity, but it is difficult to obtain the high through-plane thermal conductivity required for TIMs. Carbon nanotubes also have high theoretical thermal conductivity, but extensive results from the literature have demonstrated that the TIMs prepared using carbon nanotubes as fillers fail to reach the expected thermal conductivity due to a number of reasons, such as defects, interfacial thermal resistance, and purity. In comparison, CF is more suitable as a thermally conductive filler because it has many advantages, including high thermal conductivity, a high strength-to-weight ratio, a low thermal expansion coefficient, low cost, and excellent corrosion resistance. For example, Ma et al. [23] reported a thermally conductive TIM based on the construction of three-dimensional (3D) CF skeletons, achieving a through-plane thermal conductivity of 2.84 W·m^−1^·K^−1^ at a filling fraction of 13.0 vol%. Zhang et al. [24] reported a novel CF@SiC filler with a core–shell structure via coating a layer of SiC on the surface of CF, which was further combined with polydimethylsiloxane (PDMS) to prepare TIMs, and the composite thermal conductivity reached ~4.0 W·m^−1^·K^−1^ at a filling fraction of 55 wt%.

The traditional method to enhance the thermal conductivity of TIMs is to increase the filling fraction as much as possible, but this will lead to an increase in production cost and a decrease in the processability of composites. Therefore, rational designs of the thermally conductive network for enhancing heat dissipation efficiency has received more and more attention. The use of specific processing techniques such as vacuum filtration [25,26], freeze casting [27,28], magnetic field orientation [29], and hot pressing [30] to promote the directional alignment of fillers is a common method for constructing ordered thermally conductive pathways. Among them, the freeze-casting method utilizes the directional growth of ice crystals to drive filler alignment in the freezing direction, resulting in a 3D-oriented network structure. The freeze-casting method is very advantageous for preparing the TIMs due to the simple preparation process, good mechanical strength, controlled porosity, and the desired through-plane thermal conductivity. On the other hand, the interfacial thermal resistance is also an important factor affecting the thermal conductivity of composites [31]. Constructing hierarchical micro-nano hybrid fillers (e.g., AlN@AgNPs [32], BN@ AgNPs [33], and Al_2_O_3_-GO [34]) is considered an effective strategy for enhancing composite thermal conductivity. For example, Niu et al. [32] synthesized fillers with hierarchical structures by depositing Ag nanoparticles on the surface of AlN whiskers (AlNw-AgNPs), which were further combined with nanofibrillated cellulose to prepare thermally conductive composite films. Compared with the AlNw without AgNPs, the AlNw-AgNP hybrids could improve the in-plane and through-plane thermal conductivities by 28.9% and 7.5%, respectively. This is mainly because the hierarchical structure can effectively utilize the advantages of various fillers and realize the synergistic effect between them. In particular, the bridging-link effect of nanoparticles can effectively construct a better thermal conductivity network in favor of reducing the interfacial thermal resistance between filler–filler and filler–matrix; therefore, higher composite thermal conductivity is easily obtained. The preparation of hierarchical micro-nano hybrid fillers with CF as substrate and further construction of 3D skeleton is of great significance to enhance thermal conductivity of CF-based thermally conductive composites.

Based on the above consideration, in this study, we designed and synthesized a hierarchical hybrid filler consisting of CF and needle-like ZnO nanowires (CF@ZnO), which was then used to construct the oriented aligned filler skeletons using the freeze-casting method. After impregnation with EP, the thermally conductive composites were successfully prepared. Compared with pure CF, the CF@ZnO hybrid fillers led to a significant increase in thermal conductivity. This is mainly due to the fact that ZnO nanowires acted as bridging links and reduced interfacial thermal resistance, which in turn optimized the thermal conductivity network in the composites.

## 2. Materials and Methods

### 2.1. Materials

CF powders (purity > 97%, 400 mesh) were purchased from Toray Industries, Inc., Tokyo, Japan. Zn(CH_3_COO)_2_ 2H_2_O (AR) and Hexamethylenetetramine (HMTA) were purchased from Shanghai Aladdin Bio-Chem Technology Co., Ltd., Shanghai, China. Polyvinyl alcohol (PVA, purity > 99%) was provided by Sinopharm Chemical Reagent Co. Ltd., Shanghai, China. EP and curing agents were offered by Guangzhou Gao-Yun Chemical Co., Ltd., Guangzhou, China. EP resin 862 was purchased from Hexion Chemical Co., Ltd., Columbus, OH, USA. The curing agent of EP was purchased from Guangzhou Haoyun Chemical Co., Ltd., Guangzhou, China.

### 2.2. Preparation of EP Composites with Directional CF@ZnO Skeletons

**Figure 1:**
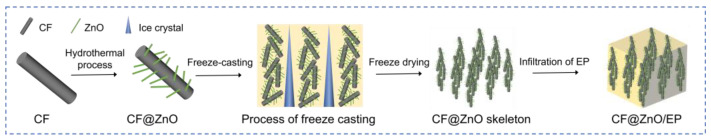
Preparation process of EP composites with directional CF@ZnO skeletons.

The preparation process of EP-based composites with directional CF@ZnO skeletons is shown in Figure 1, including three steps: (I) the preparation of CF@ZnO hybrids via the hydrothermal method, (II) the construction of directional CF@ZnO skeletons via the freeze-casting process, and (III) the preparation of CF@ZnO/EP composites via the vacuum impregnation process.

Step I: Preparation of CF@ZnO hybrids by hydrothermal method.

Firstly, CF was magnetically stirred in hydrochloric acid for 12 h to remove surface impurities and then centrifuged with deionized water to wash away the residual acid. Secondly, 0.3 g CF, 0.54 g, or 1.08 g zinc sulfate dihydrate, 0.1 g Hexamethylenetetramine (HMTA, accelerator), 1 mL ammonia (NH_3_·H_2_O), and 50 mL deionized water were homogeneously mixed, obtaining mixed solutions with a zinc ion (Zn^2+^) concentration of 0.5 mol/L and 1 mol/L, respectively. Subsequently, the solution was ultrasonicated for 30 min and then poured into a polytetrafluoroethylene container, which was further transferred into a 100 mL hydrothermal kettle. After that, the kettle was warmed up to 105 °C at 10 °C/min in a drying oven, hydrothermally treated for 20 h, and then naturally cooled. Then, the powders were centrifuged and cleaned with deionized water to wash away residual impurities, following the drying process to obtain CF@ZnO hybrids. Depending on the concentration of Zn^2+^ in the initial solutions, the prepared CF@ZnO hybrids were denoted as CF@ZnO-0.05 or CF@ZnO-0.1.

Step II: Construction of directional CF@ZnO skeletons by freeze-casting process.

The CF@ZnO powders and PVA solution (5 wt%) were homogeneously mixed, and the solid content of the slurries was controlled as 10 wt%, 20 wt%, 30 wt%, and 40 wt% by adding different amounts of deionized water. Then, the prepared slurries were poured into a PTFE mold with copper tapes at the bottom, and the mold was placed on a copper plate with the bottom immersed in −80 °C alcohol. When the slurries were completely frozen, the frozen skeletons were transferred to a freeze dryer. Finally, the CF@ZnO skeletons with oriented structure were obtained via freeze drying at −60 °C for 24 h under vacuum.

Step III: Preparation of CF@ZnO/EP composites by vacuum impregnation process.

The epoxy resin and curing agent were homogeneously mixed in a vacuum mixer at a pressure of 5.0 kPa, of which the mass ratio was 3:1. The prepared directional CF@ZnO skeletons were completely immersed into the EP resin, impregnated in a vacuum environment (~10 kPa) for 2 h, and then cured in an oven at a temperature of 60 °C for 12 h. The excess epoxy resin on the surface of the composites was removed to obtain the CF@ZnO/EP composites.

### 2.3. Characterization

The phase compositions of the CF, CF@ZnO-0.05, and CF@ZnO-0.10 were characterized at room temperature via X-ray diffraction (XRD; Rigaku SmartLab SE, Tokyo, Japan) using a Cu target, and the wavelength of the X-rays radiation was 0.154 nm. In addition, the X-ray diffraction employed continuous scanning. The step was 0.02°, the scanning speed was 10°/min, and the scanning range was 10~80°. The morphologies were investigated via scanning electron microscopy (SEM, TESCAN MIRA LMS, Brno, Czech Republic) under a voltage of 15 KeV, and the corresponding elemental distribution was analyzed using an energy dispersive spectrometer (EDS, TESCAN MIRA LMS, Brno, Czech Republic). For the sample preparation, the powder was glued directly to the conductive adhesive, and the block was glued to the sample plate using conductive adhesive, after which the samples were sprayed with gold to improve conductivity. The chemical composition and elemental states of CF, CF@ZnO-0.05, and CF@ZnO-0.10 were analyzed using X-ray photoelectron spectroscopy (XPS, Thermo Scientific K-Alpha, Waltham, MA, USA) with an excitation source of Al Kα rays (1486.6 eV). The specific characterization process was as follows: An appropriate amount of powder sample was pressed and affixed to the sample disc, which was further put into the sample chamber of the instrument. When the pressure in the sample chamber was less than 2.0 × 10^−7^ mbar, the sample was fed into the analysis chamber with a spot size of 400 μm, an operating voltage of 12 kV, and a filament current of 6 mA. The full spectrum scanning energy was 150 eV with a step size of 1 eV, and the narrow spectrum scanning energy was 50 eV with a step size of 0.1 eV. The thermogravimetric (TG) and differential scanning calorimetry (DTG) curves were measured via synchronous thermal analysis (TGA55, TA Instruments, DE, USA) at a heating rate of 10 °C·min^−1^ from 20 °C to 800 °C under N_2_ atmosphere. Thermal imaging photographs were achieved using an FTIR infrared camera (E6-WIFI, Shenzhen Yujie Hongye Technology Co., Ltd., Shenzhen, China. The thermal conductivity (TC) was measured using the laser flash method, and the TC was calculated using the following equation [35]:

TC = *α* · *ρ* · *C*_p_ (1)

where α was the thermal diffusivity, *ρ* was the density, and *C*_p_ was the specific heat capacity of the composites; the α was measured via the laser flash method using an LFA 467 (NETZSCH, Selb, Germany), *ρ* was calculated via the Archimedes method, and *C*_p_ was measured via the Sapphire method (DSC, TA SDT650) at a heating rate of 10 °C·min^−1^ [35].

## 3. Results and Discussion

### 3.1. Characterization of CF@ZnO Hybrids

**Figure 2:**
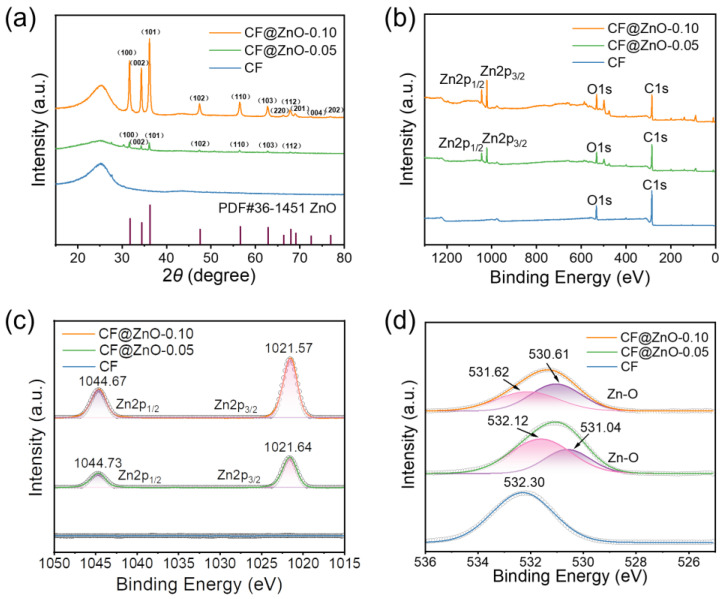
Structure and elemental characterizations of CF, CF@ZnO-0.05, and CF@ZnO-0.10 hybrids: (**a**) XRD patterns, (**b**) XPS spectra, (**c**) XPS spectrum for Zn2p, and (**d**) XPS spectrum for O1s.

Figure 2a demonstrates the XRD patterns of CF, CF@ZnO-0.05, and CF@ZnO-0.10, respectively. The peaks located between 20° and 30° are amorphous carbon, and the diffraction peaks of ZnO can be clearly seen from the samples of CF@ZnO-0.05 and CF@ZnO-0.10, indicating that the zinc sulfate dihydrate was successfully converted to zinc oxide after the hydrothermal reaction, and the reaction equations are as follows [36]:

(CH_2_)_6_N_4_ + 6H_2_O ↔ 6HCHO + 4NH_3_ (2)

NH_3_ + H_2_O ↔ NH_3_·H_2_O ↔ NH^4+^ + OH^−^ (3)

Zn(CH_3_COO)_2_·2H_2_O ↔ Zn^2+^ + 2(CH_3_COO)^−^+ 2H_2_O (4)

Zn^2+^ + 2OH^−^ ↔ Zn(OH)_2_ ↔ ZnO + H_2_O (5)

During the heating process, HMTA gradually released ammonia as an initial reactant, which is a weak electrolyte, ionizing NH^4+^ and OH^−^. Meanwhile, Zn(CH_3_COO)_2_·2H_2_O underwent hydrolysis in water to form (CH_3_COO)^−^ and Zn^2+^. Then, Zn^2+^ combined with OH^−^ to form zinc complexes and eventually converted to ZnO. Moreover, with increasing the Zn^2+^ concentration in the initial solutions, the diffraction peaks of ZnO were more obvious, indicating that the content of ZnO in the CF@ZnO hybrids improved. Figure 2b shows the XPS spectra of CF, CF@ZnO-0.05, and CF@ZnO-0.10, respectively. As observed, only two peaks of O1s and C1s were detected for the pure CF, while two obvious diffraction peaks near 1044 eV and 1021 eV appeared for the CF@ZnO-0.05 and CF@ZnO-0.10, which correspond to Zn 2p_1/2_ and Zn 2p_3/2_ (Figure 2c), respectively. Figure 2d further shows the variation pattern of O1s. The O1s peak of untreated CF was located at 532.30 eV, while the O1s peaks of CF@ZnO-0.05 and CF@ZnO-0.10 both showed a rightward shift. In other words, the binding energy corresponding to the O1s peak decreased with the increasing concentration of Zn^2+^. Fitting of the data reveals that the O1s peaks of both CF@ZnO-0.05 and CF@ZnO-0.10 corresponded to two chemical states of the O atom; one is the O atom presented in the raw CFs, and the other is the O atom in the ZnO. With the concentration of Zn^2+^ in the initial solution increasing, the area of the peak corresponding to the O atom in ZnO increased, further indicating the increase in ZnO content in the prepared CF@ZnO hybrids.

**Figure 3:**
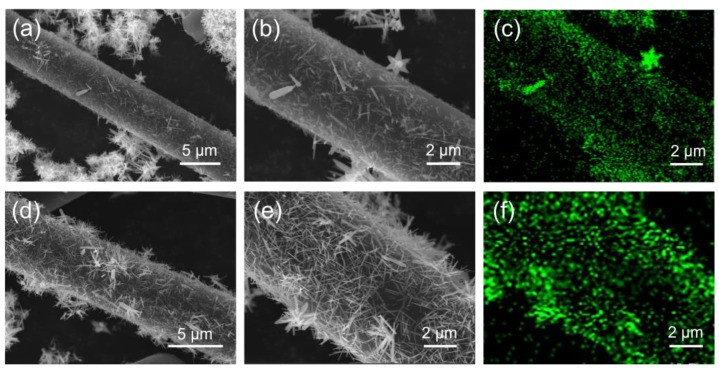
SEM and EDS mapping images of Zn element for CF@ZnO hybrids: (**a**–**c**) CF@ZnO-0.05; (**d**–**f**) CF@ZnO-0.10.

Figure 3a,b are SEM images of CF@ZnO-0.05 with different magnifications, while Figure 3d,e are those of CF@ZnO-0.10. In addition, Figure 3c,f are the distributions of the Zn elements corresponding to Figure 3b,e, respectively. As observed, needle-like ZnO was successfully attached to the CF surface after hydrothermal treatment, and the amount of ZnO increased significantly with the increasing Zn^2+^ concentration in the initial solutions. The formation of ZnO needle-like morphology is mainly due to the slow hydrolysis of Zn^2+^ during the hydrothermal process, which is consistent with the study of Liu et al. [36]. The ZnO nanowires deposited on the surface of CF constituted a hierarchical hybrid structure, which served to connect the CF to form more thermally conductive pathways, and thus, higher thermal conductivity could be expected.

### 3.2. Microstructures of CF@ZnO Skeletons and CF@ZnO/EP Composites

**Figure 4:**
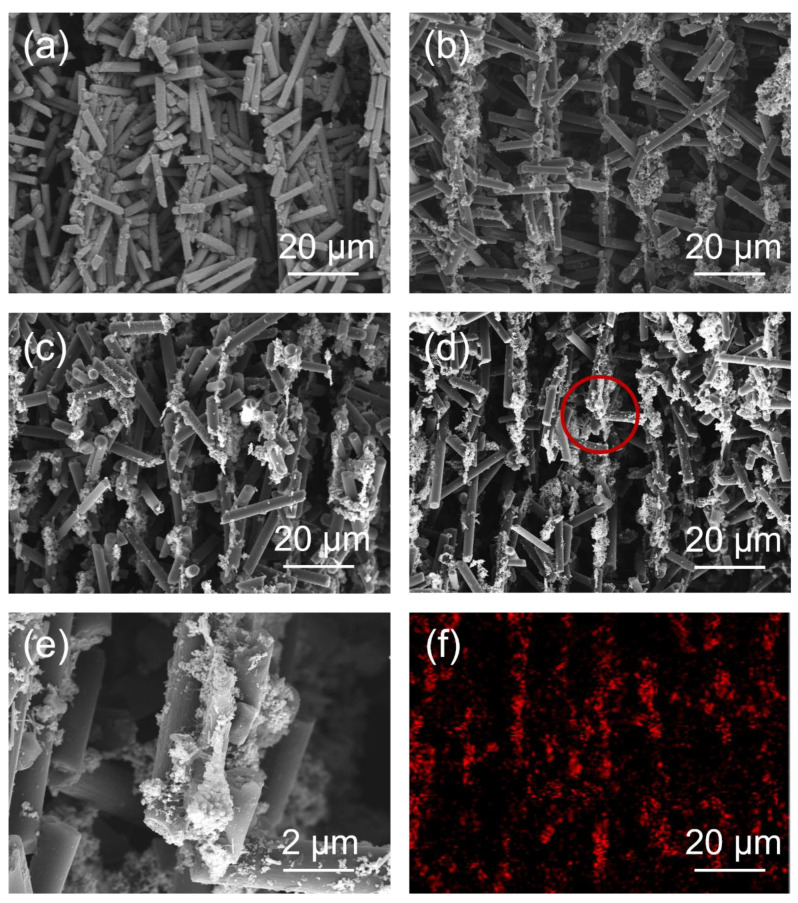
(**a**–**c**) SEM images of different skeletons prepared from slurries with 20 wt% solid loading: (**a**) CF skeleton; (**b**) CF@ZnO-0.05 skeleton; (**c**) CF@ZnO-0.10 skeleton; (**d**,**e**) SEM images of CF@ZnO-0.10 skeleton prepared from slurries with 40 wt% solid loading; (**f**) EDS mapping of Zn element corresponding to Figure 4d.

Figure 4a–c show the SEM images of CF, CF@ZnO-0.05, and CF@ZnO-0.10 skeletons prepared from slurries with 20 wt% solid loadings, from which it can be seen that the as-prepared skeletons had an obvious directional arrangement. This is mainly due to the fact that the CF skeletons were formed via the freeze-casting process. First, the prepared CF slurry was poured into a square mold and then placed into an ethanol coolant. Due to the temperature gradient, the slurry started to freeze from the bottom to up and the CF fillers in the slurry were squeezed by the ice crystals until the slurry was completely frozen. The subsequent freeze-drying process took place under a vacuum (~10 Pa, 60 °C), where the ice crystals sublimated directly into water vapor and escaped, leaving oriented voids, while the billet retained its skeleton with an oriented structure without collapsing. As a result, the three-dimensional oriented CF skeletons with pore channels arranged from bottom to top were retained [37,38]. Figure 4d–f further show the SEM images and EDS mapping of Zn element for CF@ZnO-0.10 skeletons prepared from slurries with 40 wt% solid content. The distance between the channel walls became narrower with the increasing solid loading. This is mainly because a high solid loading implied that there was less water in the initial slurry, and therefore the volume of the pore channel left behind by the sublimation of ice crystals decreased, i.e., the density of CF fillers in the skeleton increased. In addition, it can be clearly observed that the ZnO was uniformly distributed on the surface of CF after the freeze-casting process, which could improve the contact area between the CF. As a result, the CF@ZnO skeletons can provide more thermally conductive paths compared with that of pure CF skeleton, which was favorable to improve the thermal conductivity of the composites.

**Figure 5:**
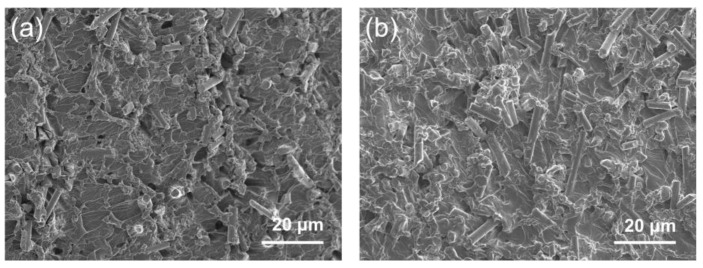
SEM images of CF@ZnO-0.10/EP composites prepared from slurries with different solid loadings: (**a**) 20 wt%, (**b**) 40 wt%.

Figure 5 shows the cross-sectional SEM images of various CF@ZnO-0.10/EP composites, which were prepared from initial slurries with 20 wt% and 40 wt% solid loadings, respectively. It can be observed that the cutting surface of composites was relatively smooth and flat, meaning that EP fully filled the pore channels in the CF@ZnO skeletons. It can be indicated that the CF@ZnO skeleton was well combined with the EP matrix. Since the thermal conductivity of EP is much higher than that of air, the complete filling of the pore channels with EP is of great interest in improving the thermal conductivity of the composites. In addition, the amount of CF exposed in the composites significantly increased as the solid loading of the initial slurries increased from 20 wt% to 40 wt%. This is because a higher CF solid loading in the initial slurry means a higher filling fraction of CF in the composites, which facilitated the formation of more thermally conductive channels by lapping between CF.

### 3.3. Thermal Performance of CF@ZnO/EP Composites

**Figure 6:**
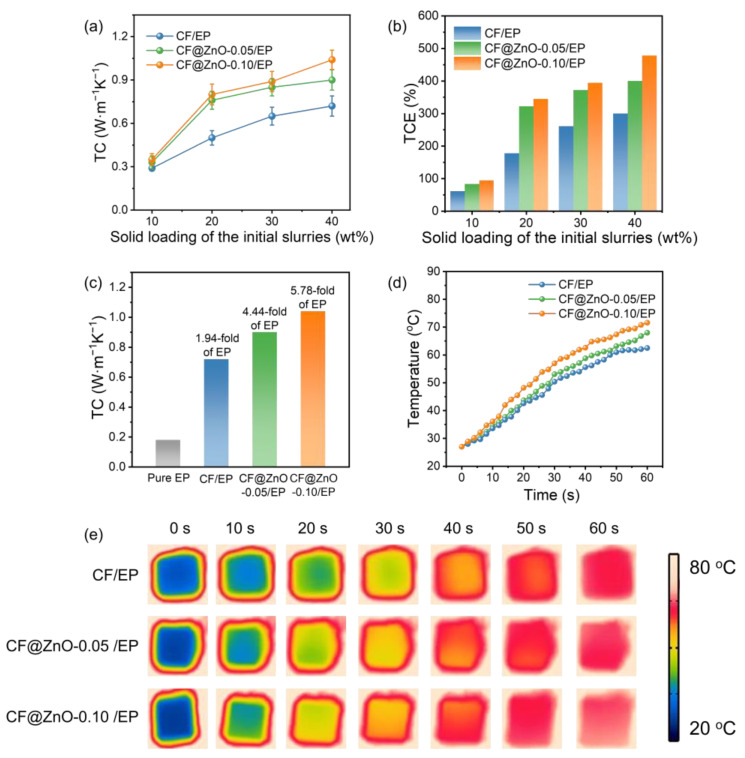
Thermal performance characterization of CF/EP, CF@ZnO-0.05/EP, and CF@ZnO-0.10/EP composites: (**a**) thermal conductivity, (**b**) TCE, (**c**) comparison of the thermal conductivity of pure epoxy and various composites; (**d**) surface temperature changes; and (**e**) infrared thermography of various composites during the heating process.

Figure 6a shows the thermal conductivity of CF/EP, CF@ZnO-0.05/EP, and CF@ZnO-0.10/EP composites prepared from slurries with different solid loading. As observed, the composite thermal conductivity gradually increased with the increasing solid content in the initial slurries, which is mainly due to the larger number of filler particles providing more thermally conductive pathways. In addition, the thermal conductivity of various composites obeyed the following order: CF@ZnO-0.10/EP > CF@ZnO-0.05/EP > CF/EP. Figure 6b further shows the thermal conductivity enhancement (TCE) of various composites, which is described as follows:

(6) $$TCE=\frac{\left(K_{c}-K_{m}\right)}{K_{m}}\times 100\%$$

where *K_c_* and *K_m_* are the thermal conductivity of composites and pure EP matrix (~0.18 W·m^−1^·K^−1^), respectively. The change pattern of TCE was similar to that of thermal conductivity, further demonstrating the beneficial effect of filling fraction and ZnO nanowires on the composite thermal conductivity. Figure 6c further compares the thermal conductivity of pure epoxy and various composites obtained from initial slurries with 40 wt% solid loading. The CF@ZnO-0.10/EP exhibited the highest thermal conductivity of 1.04 W·m^−1^·K^−1^, which was 1.44 times higher than that of CF/EP (0.74 W·m^−1^·K^−1^), and 5.78 times higher than that of pure epoxy resin (~0.18 W·m^−1^·K^−1^). The above results indicate that the construction of needle-like ZnO nanowires on the surface of CF has a positive effect on the improvement in the thermal conductivity. EP, as one kind of polymer, usually has low thermal conductivity, which is due to the strong phonon scattering from long molecular chains. CF has much higher thermal conductivity than EP, and its addition to EP could significantly increase the thermal conductivity of the composites. However, due to the large interfacial thermal resistance between CF and polymers, the thermal conductivity enhancement was not that obvious. On this basis, when a small amount of needle-like ZnO nanowires was deposited on the CF surface as the second phase, the ZnO nanowires could effectively act as bridging links between CF particles and provide more thermally conductive paths, thus forming more heat conduction pathways and promoting the reduction in interfacial thermal resistance. As a result, the thermal conductivity of the composites was significantly enhanced. Furthermore, with the quantity of ZnO nanowires increasing, more thermally conductive pathways could be formed; thus, the enhancement effect on the composite thermal conductivity became more obvious.

To further evaluate the actual thermal management performance of the EP-based composites, the CF/EP, CF@ZnO-0.05/EP, and CF@ZnO-0.10/EP composites with the same filling fraction were placed on a heating plate at 80 °C, and the temperature changes on the upper surface of various samples at different times were recorded using an infrared thermography camera, as shown in Figure 6d,e. As observed, the sample of CF@ZnO-0.10/EP exhibits the fastest heating rate, while the CF/EP shows the slowest one. The temperature change trend of various composites was consistent with the composite thermal conductivity relationship shown in Figure 6c. After heating for 60 s, the upper surface temperature of the CF@ZnO-0.10/EP arrived at ~71.6 °C, which was ~9.1 °C higher than that of the CF/EP (~62.5 °C), further demonstrating that the introduction of needle-like ZnO increased the heat conduction paths in the composites and hence provided superior ability of heat transfer.

**Figure 7:**
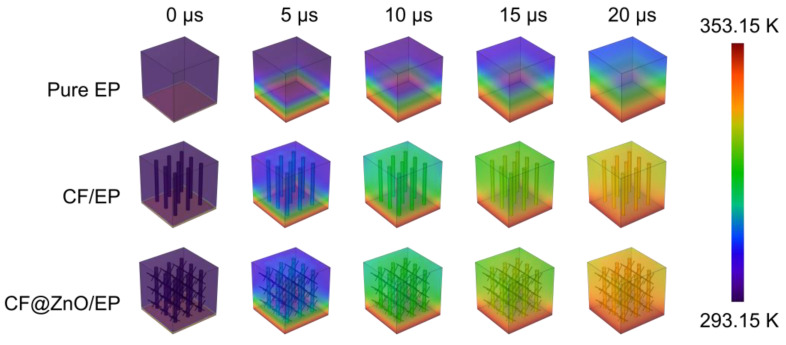
Simulated surface temperature variation with time of pure EP, CF/EP, and CF@ZnO/EP composites.

Finite-element simulation is effective in investigating the heat transfer process of polymer-based composites. In order to clearly demonstrate the thermally conductive mechanism, we further model and simulate the heat transfer process of various composites. Three cubic models with a side length of 7 μm were constructed, namely pure EP, CF/EP, and CF@ZnO/EP. A cylinder with a diameter of 0.5 μm and a height of 6 μm was created as the CF, and a cylinder with a diameter of 0.1 μm and a height of 2 μm was created as the ZnO nanowires. The materials used were all from the material library included in the software. Then, the initial temperature was set to 293.15 K while the bottom heating temperature was set to 353.15 K. The temperature change on the surface of the three models was calculated when the heat transfer time was 20 μs, and the step size was 5 μs. The simulated heat transfer process of various samples is shown in Figure 7. Compared with pure EP, the composites filled with CF and CF@ZnO had higher surface temperatures at the same time, suggesting a faster heat transfer rate. Moreover, CF@ZnO/EP was more advantageous in heat transfer compared to CF/EP, further indicating the ZnO nanowires could act as bridging links and, in turn, optimize the thermal conductivity network in the composites.

**Figure 8:**
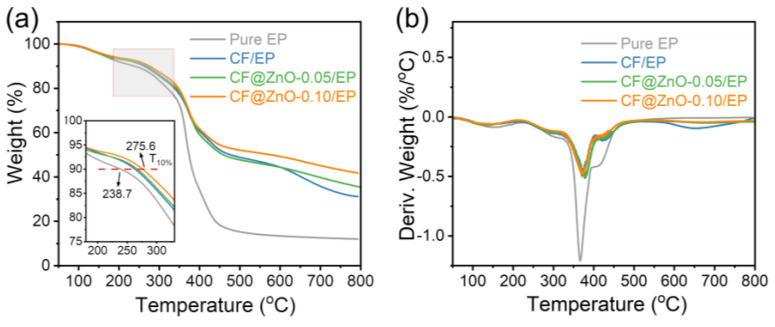
(**a**) TG and (**b**) DTG plots of the pure EP, CF/EP, CF@ZnO-0.05/EP, and CF@ZnO-0.10/EP.

Figure 8 shows the TG and DSC plots of the pure EP and the composites of CF/EP, CF@ZnO-0.05/EP, and CF@ZnO-0.10/EP composites prepared from the initial slurries with the 40 wt% solid loading. The temperature at which the mass is reduced by 10% (T_10%_) is usually used to measure the thermal stability of the composite [39]. Compared with the pure EP, the T_10%_ of the composites with CF and CF@ZnO fillers significantly shifted to the right; in particular, the T_10%_ of CF@ZnO-0.10/EP reached 275.6 °C, which was 36.9 °C higher than pure EP (238.7 °C). In other words, the temperature where the samples lost 10% of their mass was increased, which can conclude that the thermal stability of the composites was significantly improved. Figure 8b shows the mass loss rate of the samples with increasing temperature, from which the decomposition of the samples at high temperatures can be obtained. As observed, all of the samples had a prominent peak between 350 °C and 400 °C, which was mainly due to the decomposition of the EP resin at high temperatures, generating gases to escape, resulting in a decrease in the mass of the samples. With the addition of fillers, the peak values in this temperature range significantly decreased, suggesting that the fillers could slow down the thermal decomposition of EP and improve the thermal stability of the composites.

## 4. Conclusions

In this study, CF decorated with needle-like ZnO nanowires were prepared via the hydrothermal method, and the oriented alignment of CF@ZnO skeletons was realized using the freeze-casting method. After that, the CF@ZnO/EP composites were obtained via vacuum impregnation in EP resin. When the solid loading of initial slurries was 40 wt%, the thermal conductivity of the as-prepared CF@ZnO/EP composites reached 1.04 W·m^−1^·K^−1^, which was 1.44 times higher than that of pure CF under the same filling fraction and 5.78 times higher than that of pure EP. The enhancement of thermal conductivity is mainly because the ZnO nanowires acted as bridging links to increase the thermally conductive pathways and reduce the interfacial thermal resistance. In addition, the addition of CF@ZnO was also beneficial in enhancing the thermal stability of the composites, presenting wide applications in the field of heat dissipation.

## Data Availability

The data presented in this study are available upon request from the corresponding author.
